# How academic sabbaticals are used and how they contribute to research – a small-scale study of the University of Cambridge using interviews and analysis of administrative data

**DOI:** 10.12688/f1000research.74211.2

**Published:** 2023-03-14

**Authors:** Becky Ioppolo, Steven Wooding

**Affiliations:** 1 Bennett Institute for Public Policy, University of Cambridge, Cambridge, CB3 9DT, UK; 2 Research Strategy Office, University of Cambridge, Cambridge, CB2 1TN, UK; 3 Graduate School of Education M428, University of Western Australia, Perth, Western Australia, WA 6009, Australia

**Keywords:** Sabbatical, research productivity, idea origin, universities, idea generation, research on research, teaching research nexus

## Abstract

**Background**: Academic sabbaticals are seen as an important aspect of academic life and require considerable resources, however, little research has been done into how they are used and whether their effects can be measured. We explored these issues at the University of Cambridge.

**Methods**: A mixed method approach including 24 interviews with academics, eight interviews with administrators; alongside analysis of administrative and publication data between 2010 and 2019.

**Results**: Academics underline the importance of sabbaticals in providing uninterrupted time for research that is used to think, explore new ideas, master new techniques, develop new collaborations, draw together previous work, set work in a wider context, and provide personal discretion in research direction. They also highlight sabbaticals’ contributions in allowing the beneficial effects of combining teaching and research, while mitigating some of the disadvantages. However, it is difficult to detect the effect of sabbaticals on publications using a time series approach.

**Conclusions**: Sabbaticals provide manifold contributions to academic research at the University of Cambridge; however, detecting and quantifying this contribution, and extending these findings requires wider and more detailed investigation.

## Introduction

Sabbaticals are a common feature of academic work at universities in many countries around the world, but they can be misunderstood by those outside the sector ( Smith, 2020). Sabbaticals are a form of paid leave for academics in ongoing positions at established universities. By tradition, sabbaticals are earned at a rate of one term of sabbatical for every six worked. The term originated from the Greek, via Latin ‘Sabbaticus’: referring to the sabbath; and Leviticus 25:3-5 explains that the fields should be left fallow every seventh year: “but in the seventh year there shall be a sabbath of complete rest for the land” ( Kee, 1993). During sabbatical leave, an academic is usually relieved of duties related to teaching and administration. The stated reason academics have traditionally received sabbatical entitlements is to advance their research. Traditionally, sabbaticals provide the time for extended periods of travel to other institutions and allow academics to immerse themselves in reading, writing, gaining inspiration. As academic publications are the primary output of academics, it is a reasonable to assumption that sabbaticals should increase publication output.

Sabbaticals require significant resource: an academic’s teaching and administrative responsibilities must be taken over by other staff or backfilled through recruitment. Teaching is a key revenue-generating activity for most universities, and sabbaticals reduce the amount of teaching an institution can carry out - a particularly acute problem in times of austerity and financial constraint. This makes it all the more surprising that sabbaticals have not been systematically examined.

Academic literature investigating sabbaticals is very thin. The majority of articles we identified in our initial literature searches were reflective, editorial opinion pieces providing subjective, first-person advice on how to approach and make the most of a sabbatical. We were unable to find papers which systematically evaluated the cost, benefit, or efficiency of sabbaticals as a form of advancing research at the institutional, national or international scale.

This paper shares a selection of findings from a larger research project we conducted ( Ioppolo & Wooding, 2021), which studied the ways one of the world’s leading research universities – the University of Cambridge – uses discretionary funds to support research. We found that a key way discretionary funds support research is through allowing sabbaticals, which is the sole focus of this paper. The data we collected for this investigation of sabbaticals was also used for other sections of our project and other aspects of the broader project influenced how we considered the value of sabbaticals. For example, our project’s advisory group suggested the importance of investigating the interrelations between teaching, research and sabbaticals. To carry out this investigation, we were provided with access to internal administrative data sources, including sabbatical dates, grant holding, and publication records.

## Literature review

Before outlining our approach to studying the use of sabbaticals at the University of Cambridge, we conducted a light touch literature review to set our evaluation of sabbaticals into the context of any pre-existing research with similar aims.

We used Dimensions – a research documents database created by Digital Science ( Williams, 2018) – to identify articles containing “sabbatical” in the title or abstract (1,024 articles). We ranked this list by ‘FCR’ (field normalised citation) and examined the top 100 articles. We ranked the same list by the ‘Relevance’ metric provided by Dimensions and examined the top 100 articles. Six articles appeared in both lists, so a total of 194 titles and abstracts were reviewed. For a list of these articles, see Extended data ( Wooding & Ioppolo, 2021). Of these 194 articles, only nineteen contained relevant topics and were reviewed in more depth. Of these nineteen articles: three could not be sourced in full text, and eight were older than ten years old (i.e., publication date of 2009 or earlier). Therefore, the eight articles less than ten years old were reviewed in full. Of these eight articles reviewed in full, four were op-eds which were not in scope (as we were interested in empirical studies) but provided useful context for what academics see to be the contribution of sabbaticals, described in the final paragraph of this section. The remaining four articles were relevant, broad perspective studies of academic sabbaticals.

Articles we considered to be outside the scope of our review, i.e. those that were in the list of 194 but rejected when their abstracts were reviewed, covered such areas as: (a) sabbatical use in professions that are not academic (e.g., nursing, clergy, librarians), (b) use of sabbatical as one of many ‘flexible workplace practices’, (c) academic papers for which data was collected ‘while on sabbatical’, or (d) first-person narratives of the author’s recent sabbatical.

We only identified three publications from the last ten years which were empirical studies of sabbaticals, and all of these focussed on specific aspects or styles of sabbatical. A fourth paper was a historical account of the development of sabbaticals. Of the three empirical papers, Pillinger *et al.* (2019) used a survey and interviews to understand the best practices for ‘mini-sabbaticals,’ which they classified as periods from two days to six months, that researchers spent away from their home unit; typically, though not exclusively, away from the researcher’s home institution. The authors conclude that ‘mini-sabbaticals’ can fill a gap in training capacities in the clinical science contexts they studied and may provide a positive return on investment for institutions that operate in a network. The second, Altmann and Kröll (2018) looked beyond academia and surveyed employees of public and private companies in Germany, to understand how a workplace supervisor’s support can impact employees’ intention to take sabbaticals. They identified an existing stigma against the idea of sabbaticals and counterintuitively, found that supervisors actively supportive of sabbaticals, reduce employee desire to take sabbaticals because these employees already felt that their supervisor is supportive of work-life balance more generally. The third, Carraher, Crocitto, and Sullivan (2014), reviewed the literature and created a framework to help faculty members decide whether a sabbatical would be feasible and identify the optimum timing. They reviewed five empirical studies on sabbaticals which used surveys of academics to assess the publication outputs or wellbeing outcomes associated with sabbaticals. Similarly to our literature search, Carraher *et al.* found that most of the literature “is comprised of first person, anecdotal accounts or suggestions for improving the sabbatical process” ( Carraher *et al.*, 2014). They concluded that the decision to take a sabbatical is complex, and there are many factors that affect an academic’s judgement on the feasibility of a sabbatical.

The fourth study we reviewed was a historical perspective on the use of research leave for the purpose of offsite travel for academics at the University of Cambridge ( Heffernan & Jöns, 2013). The piece describes how sabbaticals emerged in the UK (they first appeared at Harvard in the 1880s, while the first research travel from Cambridge was granted in 1919) to support international travel to, for example, witness an astronomical event or examine archaeological sites in classical empires. Over the following decades, research travel became more commonly used for visiting state-of-the-art laboratories and maintaining relationships between collaborators.

These papers show us that while there has been some empirical work aiming to understand the contribution of sabbaticals, none of this research has focussed on why sabbaticals are important or the mechanisms by which they contribute to research in universities.

In addition to these four empirical publications, we reviewed four publications from the last ten years in which researchers described their personal reflections on sabbaticals, and opinion pieces about how academics can make the best use of sabbaticals ( Erren & Nise, 2010; Ling, 2020; Smith, 2020; Yarmohammadian *et al.*, 2018). These papers covered some key themes of sabbaticals we heard about in our interviews at Cambridge: specifically, these authors mentioned that the professional obligations of academic life are relentless and that the “publish or perish” environment makes sabbaticals a necessary feature to keep publishing and maintain professional credibility. The articles also noted that while friends and family outside academia sometimes see sabbaticals as a glorified vacation, the authors of these pieces considered having the chance to “recharge one’s batteries” on sabbatical was necessary to prevent burnout.

## Methods

As previously mentioned, the data collected for this work studying sabbaticals was collected for multiple purposes as it was part of a larger project about how universities use discretionary funds ( Ioppolo & Wooding, 2021). In all areas of this larger project, wanted to ensure we maximised the diversity of our relative small sample based on demographic characteristics such as their gender, discipline or career stage; and that we minimised our discretion as researchers to bias the selection. We also wanted to recognise the diverse ways that research can be considered high-quality, such as how often it has been cited or whether it has been used to underpin impacts in wider society. Finally, we wanted to choose methods that could be used at other universities across the United Kingdom if our study were to be replicated.

### Ethics

Ethical review was provided by the research ethics review board of the Department for Politics and International Studies at the University of Cambridge. Written informed consent was obtained from all interviewees by email.

We used qualitative and quantitative methods to investigate sabbatical use and their contributions at the University of Cambridge. In this initial work, we tested a variety of approaches and evolved our research strategy during the project. Our research project was supported by an advisory group (see Acknowledgements), assembled by Research England, who met with us (virtually) four times over the course of the project.

### Subject classification

We were aware that research funding varies quite markedly between disciplines. We also wanted to ensure that whichever lens we used to capture disciplinary differences could be easily replicated by other institutions wishing to further analyse the contribution of sabbaticals. Therefore, we chose to undertake the quantitative analysis by using the Research Excellence Framework’s (REF) four Main Panels organising 34 Units of Assessment (UOAs), which have already been developed with the aim of capturing the diversity of disciplinary research practices while still trying to maintain as few units as possible ( https://www.ref.ac.uk/panels/units-of-assessment/). Cambridge submitted to 30 of the 34 UOAs, and because this project was carried out while the REF 2021 exercise was being completed, all academics at Cambridge were assigned an UOA. Main Panels ^1^ were also used as an organising framework for sampling academics across the University to interview and other strands of quantitative analysis.

### Interviews

The data used in this paper on sabbaticals served many purposes, which is why such a complicated sampling strategy was used – to maximise diversity and minimise our opportunity to bias the selection. As part of a larger research project ( Ioppolo & Wooding 2021), we identified 24 academics currently employed by, or affiliated to, Cambridge to interview through a variety of approaches. In this larger research project, we wanted to examine the potential benefits of discretionary funds to support research activities with the understanding that sabbaticals are arguably supported by discretionary research funding. We were interested in both (1) identifying high quality research and tracing back its inputs to see how it may have benefitted from discretionary funds and (2) asking a diverse range of academics how they may have benefitted from discretionary funds. In order to achieve these goals, we wanted to sample a diverse range of academics with different disciplines, genders and career stages. We also wanted to recognise the diverse ways that research can be considered high-quality, such as how often it has been cited or whether it has been used to underpin impacts in wider society. This wider project on discretionary funding (of which sabbaticals is only one part) was funded as a pilot project, meaning our intent was to choose methods that could theoretically be replicated at other UK universities.

1. We interviewed six academics who had won research-related prizes featured in Meho (2020). We specifically targeted recipients of academic awards in arts, humanities, social sciences, and multi-disciplinary fields because we wanted to test this as an approach to identify high quality research that did not rely on publication citation metrics. We identified email addresses for the identified academics by web searches, BI approached them by email and 100% of them agreed to be interviewed. The interview protocol used in this study can be found as *Extended data* ( Wooding & Ioppolo, 2021).
2. We interviewed eighteen academics with a range of disciplinary backgrounds and characteristics. The first two academics were identified through a preliminary analysis of an initial dataset to test the approach. Both the analysis and data were refined before the selection of the remaining sixteen academics through the fully stratified random process (as detailed below). These interviewees were selected from list of author-publication pairs in each discipline (using UOAs), for authors currently employed by Cambridge. We carried out the data analysis on the 5 ^th^ April 2021 selecting all publications we had recorded in our administrative database since 1 ^st^ Jan 2010 until the date of interviewee selection 1 ^st^ April 2021 – this gave us 126,449 author-publication pairs.
  For each author-publication pair, we extracted the following range of publication-based significance metrics (where available):

• Dimensions Field Citation Ratio (FCR) or Scopus citation number. FCR was preferred as it is field normalised, but it has lower coverage outside the sciences. We used Scopus raw citation number in UOAs, where this increased the number of publications for which we could obtain citation figures by over 50% (we did not have access to field normalised Scopus citation figures). We used Scopus raw citation figures for UOAs B-12, C-22, D-25, D-26, D-29, D-31, D-32 and D-33.
• Altmetric Attention Score
• The internal quality score obtained in the process used to build the University of Cambridge’s REF submission, which involved scoring of the publications by two or more other academics in the same UOA.
• Whether there was a funding acknowledgement present in either the Dimensions database, or a link to a grant in University of Cambridge’s internal databases (absent/present). For more details on the accuracy of this assessment see ( Ioppolo & Wooding, 2021).

Within a given UOA, author characteristics were stratified by gender and career stage. Gender was classified into Male/Female based on Human Resource records. Career stage was broadly classified into early (REF Early Career Researcher (ECR) status), mid (by job titles ‘research fellow’, ‘researcher’, ‘university lecturer’ and other uncommon titles), or senior (‘professor’, ‘reader’ and ‘senior lecturer’). The top three author-publication pairs for each combination of publication metrics and author characteristics were included in the sampling frame (on occasion less than three publications might be included if for some combinations of characteristics there were less than three papers). For example, we included the top three papers in Law by normalised citation by female, mid-career researchers that included an acknowledgement of external funding. We repeated this selecting the top three publications for all combinations of publication characteristics, author characteristics, and funding acknowledgement for all disciplines.

Finally, we also included all author-publication pairs for authors on publications that were included as Underpinning Research in REF2021 impact case studies. ‘Underpinning Research’ is the research on which the impacts described in an impact case study were based, for more detail see the Guidance on Submissions for the REF2021, each case study can have more than one paper cited as underpinning research.

This whole process yielded a sampling frame of 3,701 author-publication pairs. Some authors could be included more than once if, for example, a female mid-career researcher was the author of a publication that had a high normalised citation and scored highly on the internal REF review score. This increased the chance that we would select that academic to interview.

Within each of the four Main Panels, we aimed to find four interviewees: a female author of a publication with external funding, a male author without external funding, etc. Within each Main Panel’s set of four interviewees, we aimed to have at least one early-career and one mid-career researcher. Additionally, wanted to ensure that we only sampled each UOA once (a criterion we relaxed for UOA A-01 and B-12 given their size). To achieve this, we used a modified random sampling approach to select interviewees from the sampling frame. We selected random author-publication pairs from the frame but rejected them if they did not fit with the existing set of selections. For example, once we had selected an interviewee from UOA B-10, we rejected any further selections from UOA B-10.

We identified email addresses for authors by web searches and BI then approached interviewees by email. Where interviewees declined to be interviewed, we returned to the sampling frame and carried out additional selections. In comparison to the ‘prize’ interviewees described above, in this second wave of approaches, we approached 38 interviewees and had 18 acceptances; a success rate of 47%. Table 1 shows the final composition of these 18 interviewees by gender, seniority, funding acknowledgement and discipline.

**Table 1.** Composition of stratified interview sample.

Interviewee sample		
Gender	Female	7
	Male	11
Seniority	Early	2
	Mid	9
	Senior	7
Funding acknowledgement	Yes	8
	No	10
Disciplinary spread		
Units of assessment		
Panel A	5	A-01, A-01, A-04, A-05, A-06
Panel B	4	B-08, B-11, B-12, B-12
Panel C	5	C-13, C-14, C-16, C-17, C-21
Panel D	4	D-25, D-26, D-29, D-33

Interviews were semi-structured and conducted via Zoom. They typically lasted 60 minutes as we spoke about a range of issues beyond sabbaticals. Conversations were recorded and computer transcribed with Descript. Transcripts were uploaded to ATLAS.ti v9 and coded for key themes by BI, using an evolving set of themes initially derived from background reading.

We also consulted University administrators, including staff from the Research Office, HR personnel, and School Finance Managers. These interviews were largely unstructured and provided us with valuable insights into local policies and practices which are not standardised across the University, and which academics may not themselves be fully aware of; however, these insights were largely about the administration of sabbaticals rather than their content and contribution to research.

### Administrative and publication data analysis

We used administrative information from the University of Cambridge to collate the start and end dates of every sabbatical taken by University academics from 1 ^st^ January 2010 to 31 ^st^ December 2019. This encompassed 2,362 sabbaticals taken by 1,319 researchers, including 1,043 pairs of sabbaticals where we can determine the gap to a subsequent sabbatical. We matched the person identifiers on these sabbaticals with administrative publication records to identify all publications by sabbatical takers over the same time period at total of 61,113 publications. These records included information on the dates and types of publication for each publication. Numerical analysis was carried out using R v3.6 ( R Core Team, 2021).

We examined the five most common publication types (representing 97% of publications recorded): articles, books, book chapters, working papers and conference presentations. As these publication records underpinned constructing Cambridge’s March 2021 REF2021 submission, these records were likely to be largely complete for the publications considered most important in each discipline (for example, conference presentations are likely to be most complete in engineering and computer science).

This analysis of publication rate was complicated by two factors. First, we needed to normalise for how frequently researchers take sabbaticals and hence how often they spend in each period after sabbatical. Second, we needed to correct for the fact that for publications where the publication date was only specified as a year, our data source recorded them as being published on the 1 ^st^ January of that year, over stating the number of January publications.

*Sabbatical frequency*

**Figure 1.**
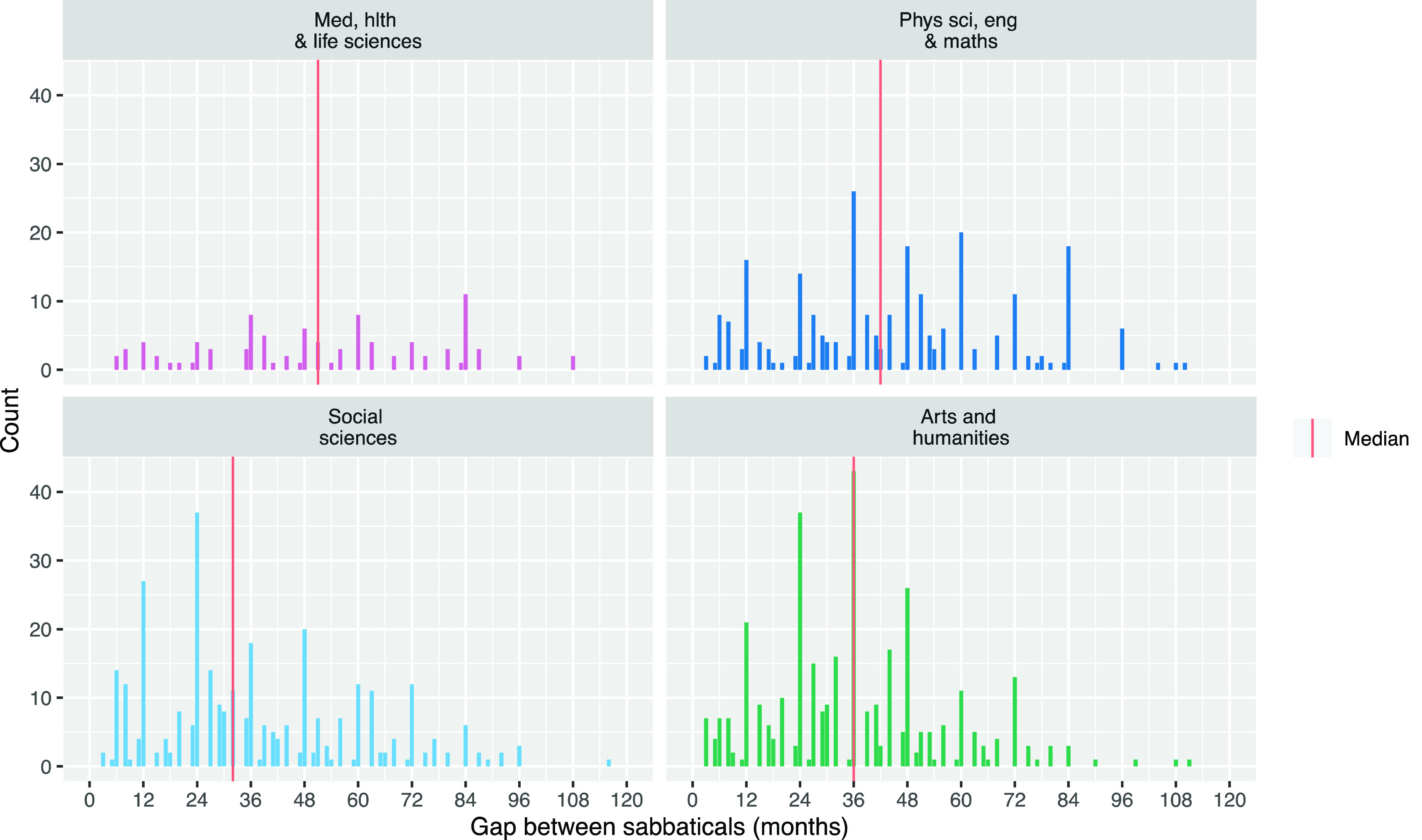
Gaps between sabbaticals taken by University of Cambridge researchers between 2010 and 2019 by Main Panel.

Considering the sabbatical frequency issue, Figure 1 shows the gap in months between researchers’ sabbaticals by Main Panel. This analysis shows there are researchers who take sabbaticals sooner than two years (six terms) after their previous sabbatical, so we cannot limit our analysis to the first two years after a sabbatical, assuming no more sabbatical time will be taken.

**Figure 2.**
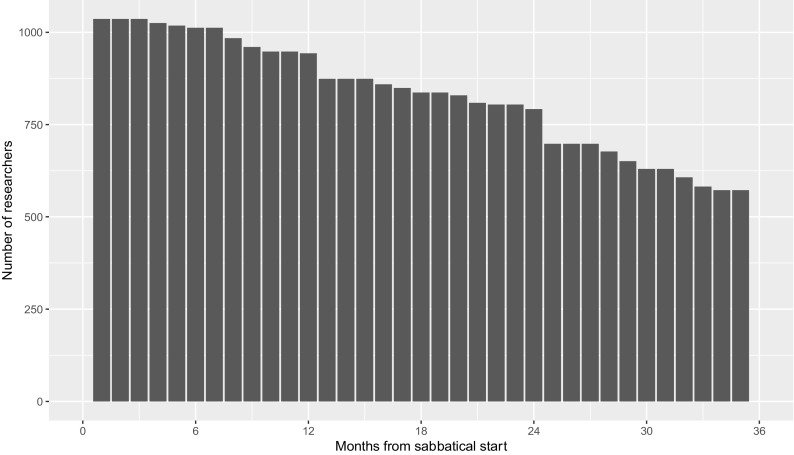
Prevalence of researchers post-sabbatical start date, by month. The graph shows the number of times each researcher has spent in each month post-sabbatical start date using data from sabbaticals taken between 2010 and 2019.

This means we have to account for how often researchers are in each period after a sabbatical. For example, in the extreme case, if every researcher took a sabbatical every year, then no researchers would ever be in their second year after their sabbatical. We would therefore expect to see no publications in the second year after sabbaticals; not because researchers were unproductive but because there were no researchers in this situation to publish papers. In reality, as shown in Figure 1, researchers take sabbaticals at varying intervals. The net result is that there are more instances of researchers in the first year after their sabbatical than there are researchers in the second year after their sabbatical. Figure 2 shows the number of times researchers are in each period after their sabbaticals in the dataset.

*Publication date accuracy – ‘the January problem’*

**Figure 3.**
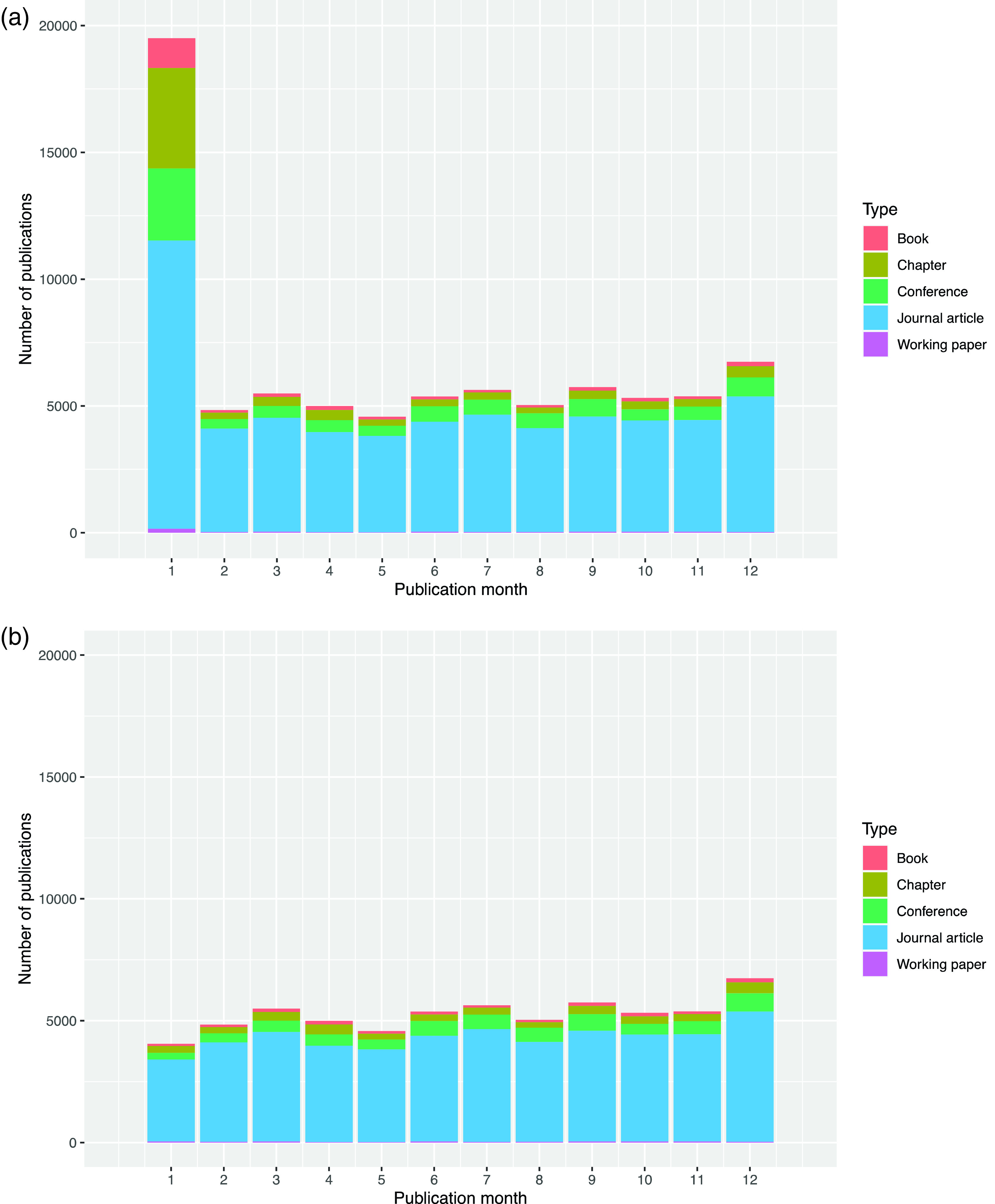
a: Publication month for all sabbatical taker publications 2010-2019. b: Publication month for all sabbatical taker publications 2010-2019 (Publications from 1 ^st^ January excluded).

Considering the second issue, the data we had available on publication dates appeared to overstate the rate of publication in the January of each year, because all publications without a recorded month are recorded as being published in January. Figure 3 shows the number of publications recorded in each month of the year.

**Figure 4.**
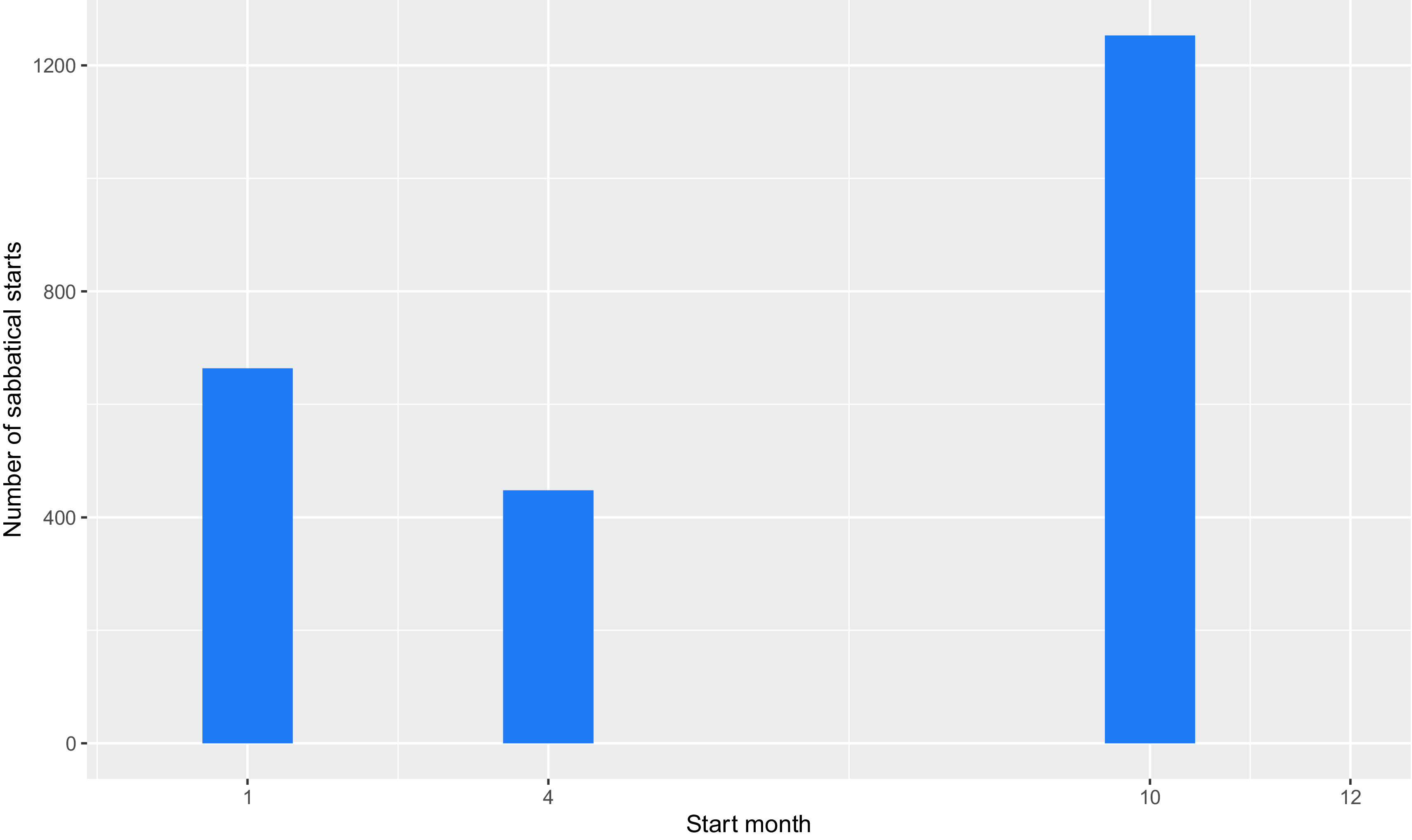
Starting month of sabbaticals (Start month of all sabbaticals taken by University of Cambridge academics between 2010 and 2019. 1 = January, 4 = April, 10 = October.

This January excess is particularly problematic, because, as shown in Figure 4, most sabbaticals begin in October. These two factors produce a false peak of publications at a delay of four months after sabbaticals start and every twelve months thereafter. We explored various ways to improve the recording of publication dates but were unable to find solution, so as a workaround, we removed all publications recorded with a date of 1 ^st^ January. This caused a slight under-representation the publication rate at four- and sixteen-months post-sabbatical.

## Results

### Analysis of sabbatical uptake

**Figure 5.**
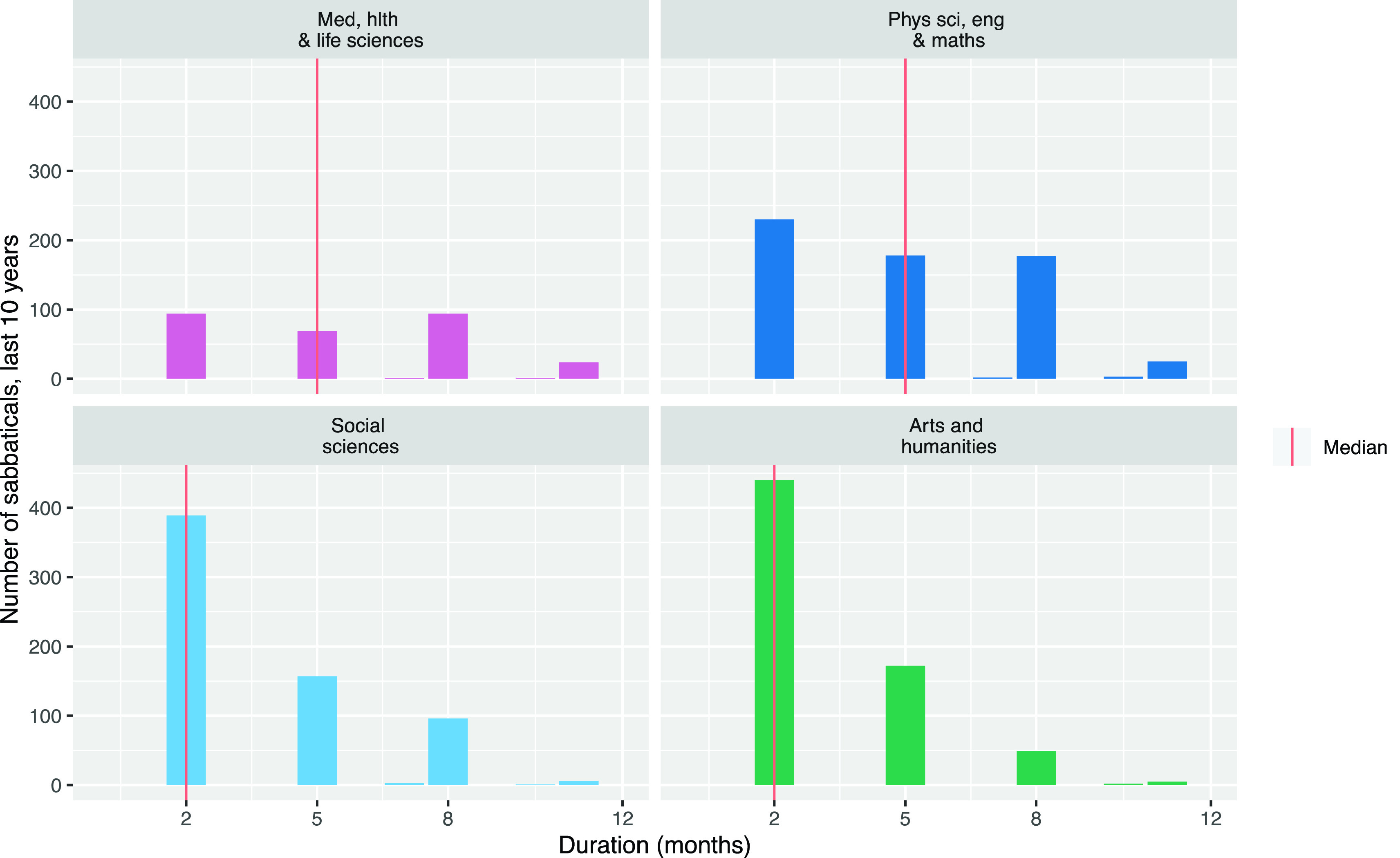
Distribution of sabbatical duration by Main Panel (Durations for all sabbaticals taken between 2010 and 2019 rounded to the nearest month).

Sabbaticals at the University of Cambridge are only available to University Teaching Officers (UTOs, tenured or tenure-track academics) and are not available to many other academic staff, for example postdocs, who are not on permanent, tenure or tenure-track posts but who nevertheless engage in teaching and research. Sabbaticals are accrued by UTOs at the rate of one term every seven and are generally taken in blocks of one to three terms (admin.cam.ac.uk, 2017) (see Figure 5).

Interviewees told us that whether academics in particular departments use their sabbaticals is heavily influenced by the departmental and disciplinary culture. These differences are clear in the quantitative data ( Wooding & Ioppolo, 2021). Sabbatical behaviour differs across the Main Panels; shorter sabbaticals are much more common in Main Panels C and D (the social sciences, arts and humanities), and in Main Panel A (clinical medicine) many sabbaticals are not taken (see Figure 5). Main Panel A includes many clinical academics who, we were told, often struggle to take sabbaticals because of the difficulty of organising cover for their clinical commitments. Our interviews with researchers and administrators confirmed that researchers have enormous freedom over how to use their sabbaticals. Although they must be approved by the Head of Department and the Faculty Board, this approval is largely an administrative process and generally not an assessment of the academic’s plans for their sabbatical.

### Summary of interviews on sabbatical contributions

We were told that the single most important aspect of sabbaticals was the provision of uninterrupted time, which academics use to think, to explore new areas, to master new techniques, to develop new collaborations and to draw together previous work or set it in a new context. This is in line with the idea that research ideas are developed more in a pattern of ‘slow hunches’ rather than in single ‘light-bulb’ moments ( Johnson, 2010). For researchers whose research consumables costs are low, sabbaticals also provide the opportunity of personal discretion and academic freedom to work on areas they judge important, even in the absence of external funding.

*Think without interruption or agenda*

The academic interviewees who had access to regular sabbaticals discussed how valuable it is to have large blocks of uninterrupted time to progress their new ideas and conduct categorically different kinds of investigation. Careful consideration of the limitations of a new idea can be challenging to do without a depth of time to allow for wandering thoughts. There may not be a plan as to where the investigation should travel. This can make it challenging to progress a new research idea with discrete one-hour blocks of time between teaching and administrative responsibilities.

One academic explained:

“[I can’t normally do these activities unless I’m on sabbatical] because I don’t get uninterrupted spells of time to think. My day is just broken up into one-hour or two-hour slots of doing stuff, and it’s very targeted. The value of just uninterrupted time to read and go follow down rabbit holes and expand…I don’t have to be strategic in how I’m deploying my time. [Being on sabbatical] felt like it is like being a PhD student again, where research is fantastic.”

Another researcher told a similar story about the importance of intellectual wandering:

“You have time where you can just sit down and think for a while. I read a bit, then I see something I don't understand so I look it up, then I see something else that's kind of interesting, so I read that too. [Sabbatical is] having the time to do that. Just following, you know, following a path, and all of a sudden it leads to a new idea. You go on a sort of walk, more instinct-driven than anything else.”

*Explore new areas and master new techniques*

A second use for uninterrupted time was to follow up those initial ideas and develop new expertise of the necessary background in a subject to pursue further funding. One researcher told us about how they had an insight about a way to bring the concepts of intellectual property into anthropology. Their sabbatical enabled them to learn the necessary background to understand and conceptualise that link, which then allowed them and others to build new areas of knowledge. In another discipline, a researcher noted that often in the early stages of developing a new research idea, it would be impossible to get external funding because the idea is still forming. They can’t even explain it to themselves at that point.

This use of sabbaticals to explore new areas also encompasses the use of sabbaticals to develop new research skills or expertise, which often provide the foundations for new projects. An example of this came from a researcher who used a sabbatical to transition from observational fieldwork to a laboratory-based experimental research portfolio. Although they had a grant to fund the set costs of the laboratory, they explained how it was crucial to have the sabbatical time alongside this to allow them to concentrate their time to set up the new laboratory.

A fourth academic highlighted the importance of being able to change direction – using a literary sporting analogy – and the importance of sabbaticals for doing so:

“[The ability to change direction is really important for research] it’s often supposed that there’s a kind of unrolling carpet: The pathway is known in advance and there are just hurdles to clear. The fact is that certainly in my field, and speaking as a historian of science, I think in most fields, research isn’t like hurdles, it’s more like Alice playing croquet [where the hoops keep moving]. Without a sabbatical, you simply cannot start a new project. Because you don’t have the time, at least in the humanities, to read around a relatively unfamiliar field.”

Giving a personal example, this interviewee explained how sabbatical leave (combined with the ‘sabbatical equivalent’ teaching and administrative buyout of a fellowship) allowed them to prepare the groundwork for a project that became a five-year grant from the Arts and Humanities Research Council to catalogue and digitise the records of the Board of Longitude. This resource is now one of the most visited sites in the University of Cambridge digital library and led to a raft of publications and meetings. The academic was clear that preparatory work done during the sabbatical and fellowship was central to the success of the project:

“It was a big project, the biggest I’d ever been involved with and very successful. [It would have been] impossible without the previous 18 months’ preparation because it was an area I’d done no work on. At all. [Prior to the sabbatical] I had no track record in this area whatsoever.”

*Develop new collaborations*

Some researchers mentioned using sabbaticals to travel abroad to do fieldwork, to allow them to work with collaborators for extended periods, and to attend conferences without teaching and administrative responsibilities requiring them to stay in Cambridge. These activities would not be possible without a period of dedicated research time like a sabbatical where distractions are limited. In this context, they also highlighted a synergy between teaching and research: having the skills to teach courses at the institution they are visiting allows academics on sabbatical to meet more people over a longer period of time (a whole term instead of a week during term breaks) and increase their chances of developing new collaborations.

*Draw together previous work*

A researcher from the humanities highlighted the importance of having time to re-immerse themselves in their wider collection of data from a range of projects:

“I think [the sabbatical] gave me the [time to] go back to my data and really ask grounded questions. [Our discipline emphasises letting] the data determine your questions rather than starting with a question. The question changes because what you find is not what you could have predicted. So, I think that the ability to go back to the data and sort of ask in an honest way, or what actually is significant about this, and that’s something that takes months, really months or years to do.”

One researcher who has not yet been eligible for sabbatical told us: “I’ve got loads of unwritten up papers where some of the analysis has been presented at a conference, but I haven’t had time to write in a journal article. So, when I get the sabbatical, I’ll try and write up some of those.”

*Set one’s work in another context*

The opportunity of focussed time can also allow researchers to frame their ideas in a wider context and present them to a wider audience. One researcher reported how two terms of sabbatical were used to write a popular science book and explained the motivation to do this:

“I wanted to write a book about [my research programme] as a synthesis of quite a big literature. And then I thought there’s no point in spending ages writing this if about 12 [experts] read it, [and] because it’s exciting and interesting to a broader audience… [The project] took longer than I expected because I’ve had to learn how to write for a different audience. But I found it incredibly rewarding, and it has transformed my teaching upon return - my teaching skills have [improved].”

*Provide personal discretion in research direction*

Where research requires little in the way of consumables, sabbaticals provide the freedom to pursue ideas that aren’t currently of interest to external funders. One researcher told us how the time allows for contribution to areas of research which are not favoured or are ‘unpopular’ for external funding but are still valuable avenues of inquiry to contribute to the discipline:

“What [external funders] tend to want to do is policy related. And there are no non-ideological answers to that, and I’m just not really that interested in going down those kinds of rabbit holes. What I want to do actually is much more reflective and ‘blue sky’: theoretical but applied to policy. There are not huge amounts of funding interest in that.”

### Summary of interviews on the teaching-research nexus

Our interviews highlighted the benefits and challenges of integrating teaching and research and how sabbaticals help mitigate the key challenge of providing a respite from the time fragmentation produced by teaching.

At the University of Cambridge, tenure-track university academics manage their own research portfolio in combination with a set of teaching responsibilities for undergraduates and post-graduate students. When we explored the reciprocal influences of teaching and research with our interviews, they highlighted benefits and tensions. This reflects an ongoing debate in the literature about whether performance in teaching and research are positively correlated, known as the teaching-research nexus ( Daumiller & Dresel, 2018; Marsh & Hattie, 2002; McKinley *et al*., 2020; Stappenbelt, 2013; Taylor, 2007).

Our interviewees noted how their involvement in teaching, at least partly allowed by their sabbaticals away from teaching, contributes to their research in a number of ways – principally by maintaining their breadth of expertise. One of the most consistent themes shared by academics across different disciplines was the belief that their teaching commitments force them to read outside their areas of research expertise. One academic spoke about feeling obligated to keep presentations to students updated with the most recent literature year on year. This led them to spend more time reading than they might otherwise do, despite recognising its intrinsic benefit. In essence, teaching “stops you from forgetting all the things you learned as a student.”

In particular, being involved in teaching requires academics to have a sufficiently deep understanding of the material in the curriculum to be able to teach it. One academic told us: “Teaching has required that I covered a much wider range of theoretical issues than I might otherwise have done, [thereby] becoming interested in those open new avenues.” Another academic mentioned that the benefits of being involved in teaching include: “it helps [me] clarify the arguments, the cause and effects, the linkages and sets up clearly in [my] mind what it is that [I am] saying.”

Academics noted that there is a distinct difference between being able to teach in more specialised topics that align with one’s research agenda compared to teaching first-year undergraduates. But in almost all cases, academics still reported that they enjoyed teaching at least to some extent and noted the benefit that the interaction of teaching helped them to feel more integrated within the University community.

Academics also felt their teaching is improved by being involved in research. They talked about how incorporating their research into the curriculum “excites the students,” “fosters creativity in the students,” and that it benefits the students to see “where knowledge is being produced actively and how it's being produced.”

As noted in the section above, academics felt that the key conflict between teaching and research was the time taken by teaching and the way teaching (and administrative) work takes time that could otherwise be used for research, one commented “sometimes it does feel like [teaching] takes time away from my research”. They particularly highlighted how teaching and administrative commitments broke up their time and took headspace away from thinking about research. Another academic noted, “I can’t think because my day is broken up into little bits.”

Given this tension, a number of our interviewees highlighted how regular sabbaticals were key to providing the combination of teaching-based inspiration and breadth, alongside opportunities for extended periods of uninterrupted thought without teaching and administrative obligations. One academic described the degree of the necessity of sabbaticals:

“I think the administrative burden on [academics] in this University has increased to such an extent that in-term research has become impossible. Where one used to be able to plan around sabbaticals, [sabbaticals are] now indispensable. Yes, without them I could not do research, and that's true for the vast majority of my colleagues… We're talking about [sabbaticals acting as] lifebelts rather than flight.”

Another academic provided this perspective on the importance of sabbaticals in providing relief from demanding teaching duties, which helps illustrate the broader effect sabbaticals can have on improving academics’ wellbeing:

“The knowledge of having a term off is great. It puts you at ease and sometimes helps in situations [of intense teaching demands] where you think: Why am I doing this? [Knowing a sabbatical is coming gives] you that light at the end of the tunnel.”

In essence, sabbaticals allow university researchers to effectively combine teaching, which maintains their exposure to a broader range of literature, individuals and ideas, often the feedstock for new ideas, with uninterrupted time to conceive and explore those research ideas.

### Analysis of publication rates

**Figure 6.**
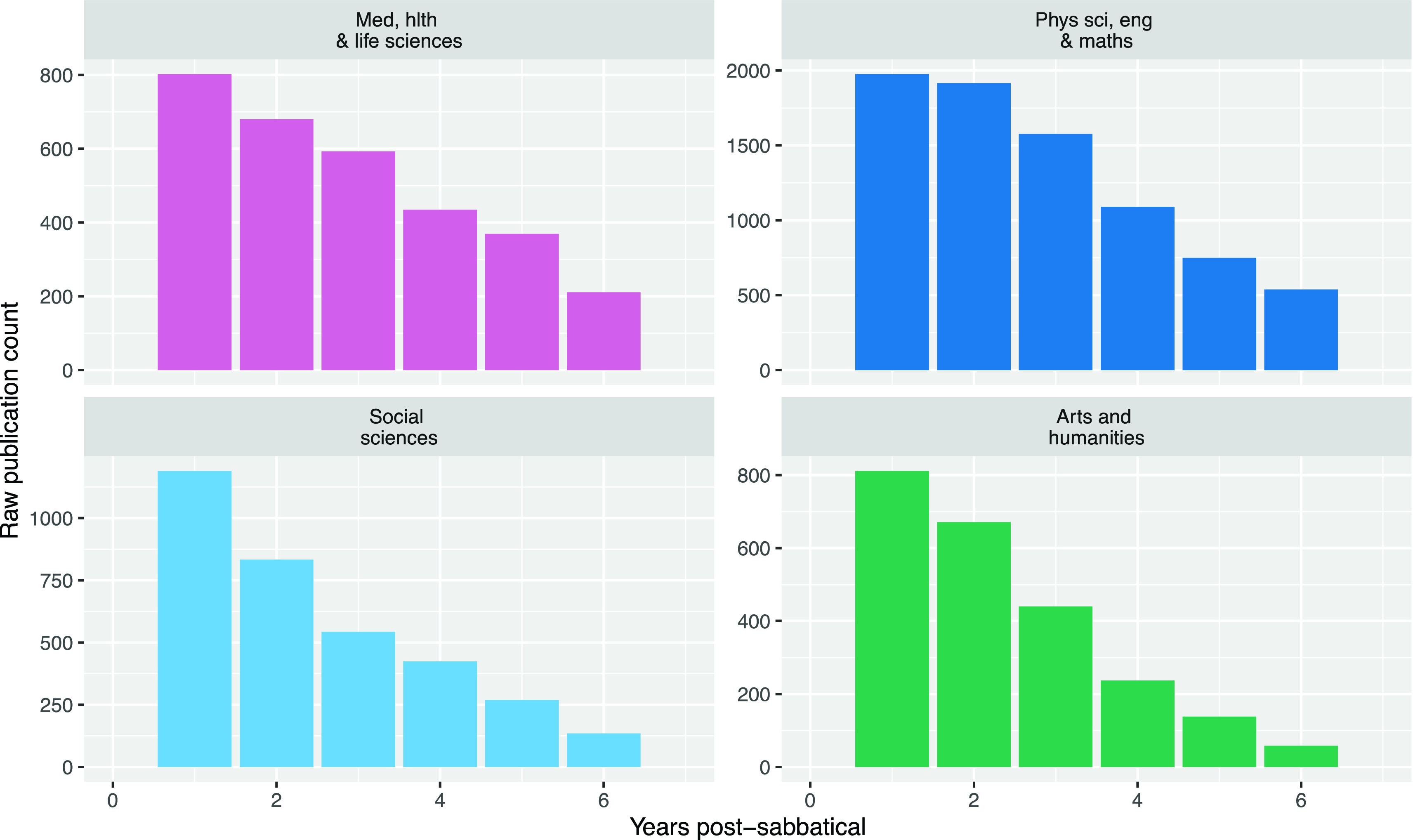
Raw publication counts post-sabbatical by year from sabbatical start (Raw publications counts in the years following the start of a sabbatical, for all researchers taking sabbaticals from 2010 to 2019).

After hearing from the interviewees about how they use sabbaticals, we combined the data on the timing of all University researchers’ sabbaticals and their publication record, to look for changes in the numbers of publication after sabbaticals. When we examined the raw publication rate post-sabbatical, on a yearly timescale (to avoid the January publication surplus issue), we saw an increase in publication rate following sabbaticals ( Figure 6). However – this effect appears to be largely due to the larger number of times researchers spend in their initial years after sabbatical (when sabbaticals are short and taken frequently), providing them more opportunities to publish at these times (as examined in the Methods section).

**Figure 7.**
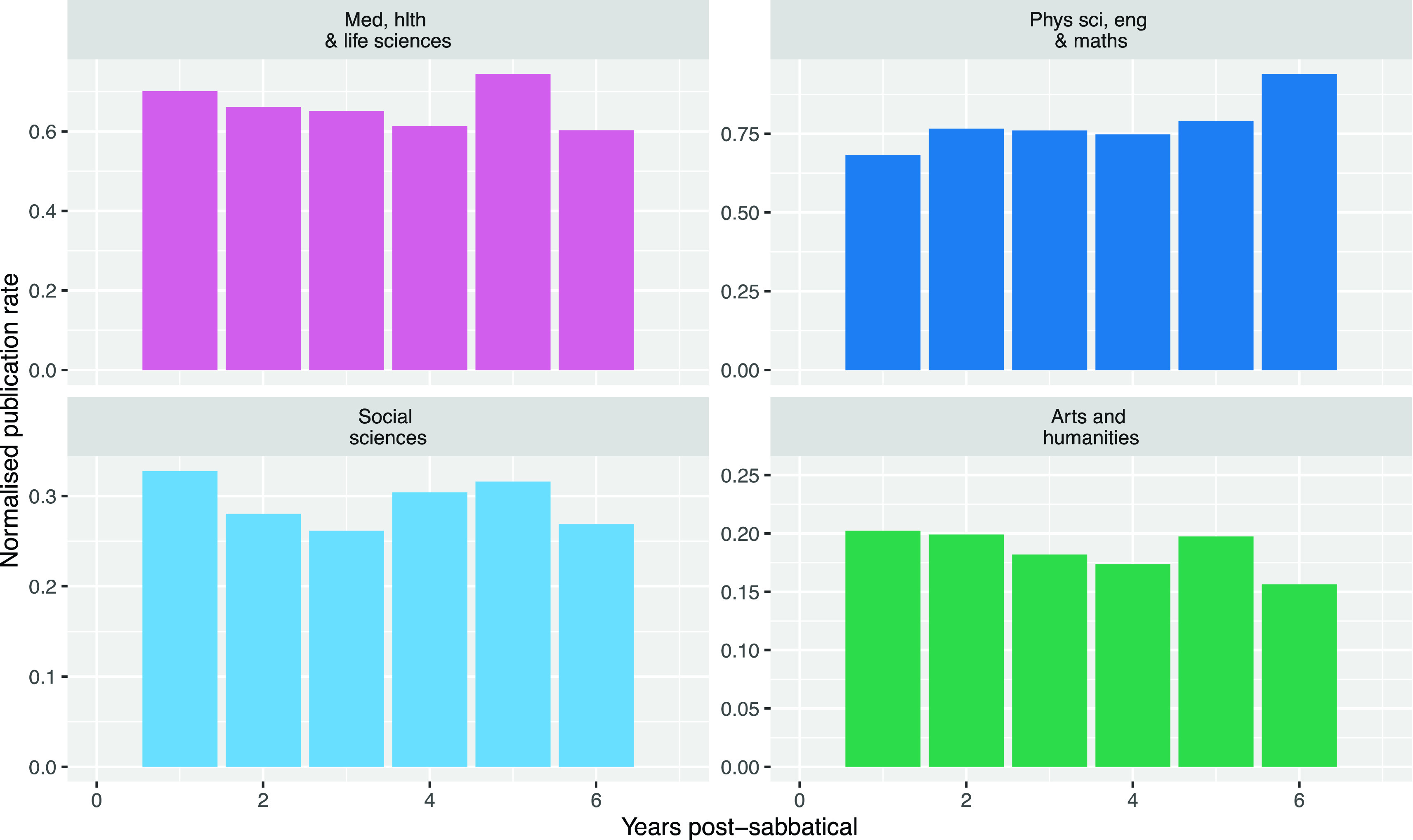
Normalised publication rate post-sabbatical by year from sabbatical start (Normalised publication rate in the years following the start of a sabbatical for all researchers taking sabbaticals from 2010 to 2019).

When we normalised publication rate by dividing the publication rate by the number of times researchers spend in each period after sabbatical, the increases in publication post-sabbatical were no longer visible ( Figure 7). Because most researchers who take sabbaticals take them relatively frequently, there are fewer researchers five or six years after their sabbaticals, so the normalised publication rates become increasingly stochastic as the time after sabbatical increases.

**Figure 8.**
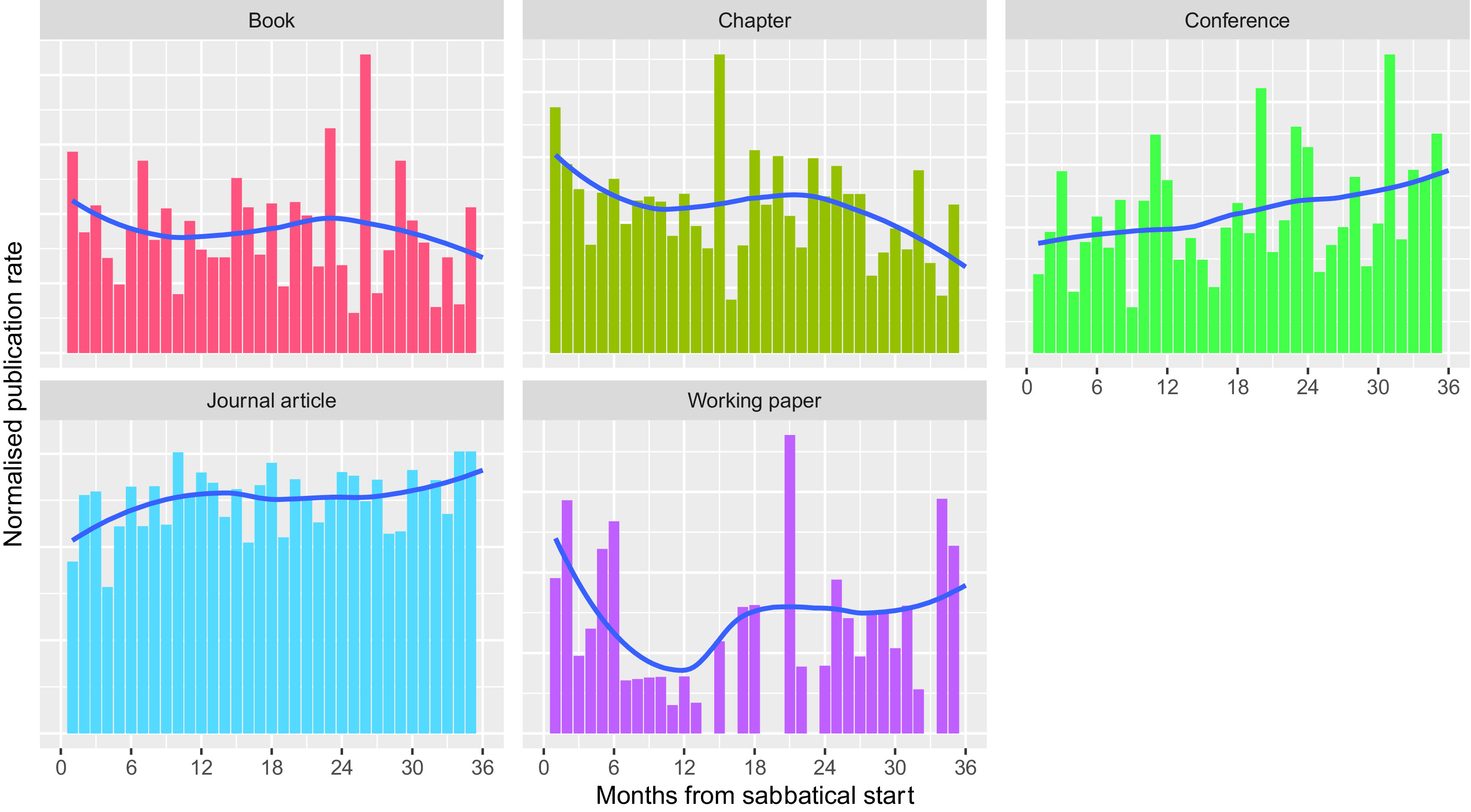
Normalised publication rate post sabbatical by month from sabbatical start – 1 ^st^ January publications excluded (Normalised publication rate in the months following the start of a sabbatical for the five most common publication types, for all researchers taking sabbaticals from 2010 to 2019. 1 ^st^ January publications excluded because of data quality concerns. Trend line for normalised publication rate calculated for each month using a local polynomial regression implemented as LOESS).

To check if there was a more rapid effect of sabbaticals, we examined the normalised publication rates on a monthly timescale while excluding 1 ^st^ January publications. In the months after the start of a sabbatical, there is a suggestion that the rate of working paper publications peaks after sabbaticals ( Figure 8). This analysis adds some weight to the idea that sabbaticals allow the generation of new ideas. There is also a suggestion of a slight increase in the rate of book and chapter publications during the months following the start of a sabbatical; however, we suspect that, if present, this does not represent new ideas, but the completion of previous work. We note, though, that this phenomenon could also be expected to apply to journal articles, however, here there might be a decreased publication rate in the first few months of sabbatical.

## Limitations

This study of sabbaticals at the University of Cambridge has a number of limitations. These findings were elicited from a broader study about how the University uses discretionary funding to support research. While sabbaticals emerged as a clear and important mechanism for supporting research via discretionary funds, sabbaticals were not the sole focus of the study. This means that there may be characteristics of sabbaticals we have missed, we did not, for example, specifically probe the ways sabbaticals are used differently by different groups of people or how policies on sabbaticals differ to other university policies.

Furthermore, this study only investigated the way sabbaticals are used at the University of Cambridge Selecting Cambridge has the advantage that there is a huge diversity, and large quantity, of research to examine; but it is also wealthy and idiosyncratic in governance and organisation in ways that could influence the practice of taking and using sabbaticals.

## Conclusions

This small-scale, mixed-methods study of the contributions of sabbaticals provide to the university environment contributes to the understudied area of sabbaticals. We found that sabbatical use varies by discipline, but the contributions that sabbaticals provide have same central themes across all disciplines.

Our interviewees make clear the importance of uninterrupted time for generating new ideas and allowing the exploration of new areas and changes of direction. We were told how uninterrupted time is also valuable to research synthesis projects and building collaborations. Our interviewees described how the sabbatical system helps square the circle of effectively integrating teaching and research. Sabbaticals provide windows of uninterrupted time for research, with the time between sabbaticals providing the intellectual benefits of teaching. Our interviewees were very clear on the contributions of sabbaticals and gave us examples of how the uninterrupted time and freedom to think “around the subject” was central to their ability to conceive significant and innovative research ideas and develop and nurture them. Often they could also link new books, research themes and papers to those sabbaticals.

However, it is much harder to detect this relationship quantitatively in research metrics. We could only identify a clear indication of a productivity increase for the (relatively uncommon) publication type of working papers. We suspect the difficulty of detecting a relationship with other publication types is because of the long and variable timescales of book publishing, and because changes at the journal article level may be more about changes in subject matter rather than changes in volume of publication. The patterns of externally funded research leave may also add noise to any signal related to sabbaticals.

Given the strongly held beliefs and compelling examples of benefits provided by researchers in our qualitative work, and the significant cost of sabbaticals, we suggest it would be beneficial to develop a better understanding of the contribution of sabbaticals. Two potential areas for further investigation are:

1) Mapping the range of sabbatical practice nationally and internationally as universities around the world will have different practices on sabbaticals and likely a different set of demonstrable benefits.
2) Exploring a set of individual cases to test whether the quantitative impact of sabbaticals can be teased out of the noise and vagaries of academic publishing processes. Other metrics could also be used to assess research productivity – we attempted initial analysis of grant applications but were unable to clean the data to an acceptable level. Additional metrics could be the number of research events (i.e., conferences, roundtables) attended or organised.

A better understanding of the contribution provided by sabbaticals could valuably inform the debate about whether we have an appropriate balance of unconstrained and pre-specified/targeted research funding.

## Data availability

### Underlying data

The interview data generated and analysed during the current study cannot be sufficiently de-identified and therefore cannot be made publicly available, due to ethical considerations. In addition, the raw bibliometric analyses data that supported the interview sampling cannot be made publicly available, due to Scopus’ and University of Cambridge’s licence agreements. The data could potentially be made available upon reasonable request, for the purpose of further research. For this, please contact the corresponding author.

However, the following data is available.

Apollo: Research data supporting [How academic sabbaticals are used and how they contribute to research – a small-scale study of the University of Cambridge], https://doi.org/10.17863/CAM.77152 ( Wooding & Ioppolo, 2021)

This project contains the following underlying data:

• Prevalence of researchers post-sabbatical start, by month.csv
• List of publications identified in the sabbatical contribution literature review.csv

### Extended data

Apollo: Research data supporting [How academic sabbaticals are used and how they contribute to research – a small-scale study of the University of Cambridge], https://doi.org/10.17863/CAM.77152 ( Wooding & Ioppolo, 2021)

This project contains the following extended data:

• Publications by type post-sabbatical start, by month.csv
• Interview protocol (final).docx

Data are available under the terms of the Creative Commons Attribution 4.0 International license (CC-BY 4.0).

## Footnotes

^1^
Main Panel A: Medicine, health and life sciences; Main Panel B: Physical sciences, engineering and mathematics; Main Panel C: Social sciences; Main Panel D: Arts and Humanities.
