# Supplementary material for: How academic sabbaticals are used and how they contribute to research – a small-scale study of the University of Cambridge using interviews and analysis of administrative data
Source: F1000Res. 2023 Mar 14;11:36. Originally published 2022 Jan 12. [Version 2] doi: 10.12688/f1000research.74211.2 (PMC10192944; doi:10.12688/f1000research.74211.2)
Supplement: Peer review history

# Peer review: Reviewer response for version 2

**DOI:** 10.5256/f1000research.145368.r166340

**Published:** 2023-05-17

**Authors:** Kathryn Oliver

**Affiliations:** 1 Faculty of Public Health and Policy, London School of Hygiene and Tropical Medicine, London, UK

Thanks, most of my comments have been addressed. I still don't understand why you have excluded first-person narratives of sabbaticals, particularly since you did interviews - but perhaps these could be written up together in a different publication.

Is the work clearly and accurately presented and does it cite the current literature?

Yes

If applicable, is the statistical analysis and its interpretation appropriate?

Yes

Are all the source data underlying the results available to ensure full reproducibility?

No source data required

Is the study design appropriate and is the work technically sound?

Yes

Are the conclusions drawn adequately supported by the results?

Yes

Are sufficient details of methods and analysis provided to allow replication by others?

Yes

Reviewer Expertise:

Evidence production and use

I confirm that I have read this submission and believe that I have an appropriate level of expertise to confirm that it is of an acceptable scientific standard.

# Peer review: Reviewer response for version 2

**DOI:** 10.5256/f1000research.145368.r166341

**Published:** 2023-03-29

**Authors:** Katherine Christian

**Affiliations:** 1 School of Arts, Federation University Australia, Ballarat, Vic, Australia

I approve these revisions and thus approve the article to be indexed.

Is the work clearly and accurately presented and does it cite the current literature?

Partly

If applicable, is the statistical analysis and its interpretation appropriate?

Yes

Are all the source data underlying the results available to ensure full reproducibility?

No source data required

Is the study design appropriate and is the work technically sound?

Partly

Are the conclusions drawn adequately supported by the results?

Yes

Are sufficient details of methods and analysis provided to allow replication by others?

Yes

Reviewer Expertise:

Mixed methods research into views of academic researchers in the sciences about their workplaces

I confirm that I have read this submission and believe that I have an appropriate level of expertise to confirm that it is of an acceptable scientific standard.

# Peer review: Reviewer response for version 1

**DOI:** 10.5256/f1000research.77940.r154953

**Published:** 2022-11-14

**Authors:** Katherine Christian

**Affiliations:** 1 School of Arts, Federation University Australia, Ballarat, Vic, Australia

I was very happy to review this paper as the topic is valuable, particularly if there is little previous research. It was also interesting to read your findings, thank you.

 I wonder if your literature search has been wide enough. It seems very surprising that you found so little literature about the benefits, or otherwise, of a practice which has been in place in academia for a very long time.

 I was surprised you went to such a great deal of trouble to select your interview participants. Was this complexity really necessary? What would have been different if, at the other extreme, you had you simply recruited 24 people from different faculties who had been on a recent sabbatical?

 It was unclear if your aim was to select people who had had publications post sabbatical, although as you went to so much trouble looking for publications, I suspect this was so. If this is so, it is not explained.

 Overall, the work is a little unclear in its presentation and would benefit from re-ordering and editing.

*Introduction*

 While logic suggests there might well be a connection between sabbaticals and increased publication output, this is not mentioned in your introduction. This link should be explained and backed up with references, if they exist, or else it could be proposed as part of your hypothesis. This is particularly important as you go on to explain, in great detail, selection of interviewees on the basis of their publication outputs.

 Somewhat related, I suggest this sentence, for example, belongs in the introduction: “Early in our project, our advisory group suggested the importance of investigating the interrelations between teaching, research and sabbaticals.”

*Literature review*

 I suggest that this be a stand-alone section ahead of methods. The section on literature review currently in Results, which unnecessarily repeats information from the earlier section, could be brought into it.

*Methods and Results*

 The Results sections, while very interesting, bears little relationship to the long explanations in Methods. I suggest the Methods should be amended to tie better to the reporting of the Results.

 You mention you also consulted University administrators, including staff from the Research Office, HR personnel, and School Finance Managers. What did they say, and/or what contribution did they make to the project?

Is the work clearly and accurately presented and does it cite the current literature?

Partly

If applicable, is the statistical analysis and its interpretation appropriate?

Yes

Are all the source data underlying the results available to ensure full reproducibility?

No source data required

Is the study design appropriate and is the work technically sound?

Partly

Are the conclusions drawn adequately supported by the results?

Yes

Are sufficient details of methods and analysis provided to allow replication by others?

Yes

Reviewer Expertise:

Mixed methods research into views of academic researchers in the sciences about their workplaces

I confirm that I have read this submission and believe that I have an appropriate level of expertise to confirm that it is of an acceptable scientific standard, however I have significant reservations, as outlined above.

# Response

**Published:** 2023-03-08

**Authors:** Steven Wooding

**Affiliations:** University of Cambridge, UK

We appreciate the interest and time of our reviewers – and their suggestion that the piece is a valuable contribution to a sparse field. Because both reviewers commented on similar themes, we have prepared a combined response which we have submitted to both reviewers. In our response to reviewers, we have organised their comments into themes and detailed how we have responded to each set of comments.

**Literature review**

*Reviewer 1: You say the literature is very thin - can you provide some examples? It would also be useful in the initial paragraphs to clearly state what the questions are which you think these papers fail to address. e.g. (guessing) They fail to evaluate the cost of sabbaticals to universities, the impact on peers, careers, research productivity, teaching participation etc.*

*Reviewer 2: I wonder if your literature search has been wide enough. It seems very surprising that you found so little literature about the benefits, or otherwise, of a practice which has been in place in academia for a very long time.*

*Reviewer 1: I see you excluded all the 1st person narratives about sabbaticals. Would these not help in contextualising your conclusions? (maybe not of course, but good to say clearly why not).*

 Both Reviewers 1 and 2 requested more explanation as to why the literature review yielded so few results which we found useful. We were similarly surprised by the absence of literature but unfortunately, we don’t have particular insights into what has caused it. We were specifically looking for studies that evaluated at a systems level (institutional, national, or international practice) whether and how the structure of a sabbatical advances research in ways that are fundamentally different to standard business as usual in universities. We wonder whether other evaluations of sabbaticals in universities have not been conducted because sabbaticals are a standard benefit of working in academia, and evaluators (academics) may fear discovering an undesirable result from their evaluations: that sabbaticals are not effective at advancing research. We already had first-person narratives about how sabbaticals are used from interviews we conducted at the University of Cambridge, which was the focus of our research. The first-person narratives discovered through the literature review did not take a broad, systems-level approach to their reflections; instead, they were summaries of project-specific actions taken during a sabbatical. We also preferred to draw on a set of reflections where we had control over the sampling rather than reflections in the literature.

 We have edited the Introduction section to clarify this.

**Interview sample and sampling strategy**

*Reviewer 1: On the interviews: I don't fully understand why you took such a complicated sampling strategy for the 18 non-prize winners. Can you explain? I assume you wanted a process to enable you to generate a sample stratified along certain criteria? What might be the downside of your approach (I'm thinking that not everyone was selected for submission to the REF for instance).*

*Reviewer 2: I was surprised you went to such a great deal of trouble to select your interview participants. Was this complexity really necessary? What would have been different if, at the other extreme, you had you simply recruited 24 people from different faculties who had been on a recent sabbatical?*

*It was unclear if your aim was to select people who had had publications post sabbatical, although as you went to so much trouble looking for publications, I suspect this was so. If this is so, it is not explained.*

 Reviewers 1 and 2 both commented that the sampling strategy seemed perhaps more intricate than necessary. The previous version of this article did not sufficiently emphasise that the research presented here about sabbaticals was part of a larger study on how discretionary funds are used in universities to support research (one way of which is via sabbaticals). The data we collected for the larger project served multiple purposes, which is part of the reason why such a complex sampling strategy was used. In this wider sampling strategy, our aim was to maximise diversity of interviewees across disciplines, gender and career stages, alongside minimising the scope for any of our biases to affect the selection. We wanted to ensure that we captured any differences in the way sabbaticals were used for researchers from these different groups.

 We have provided more context and an explanation of these reasons.

 Reviewer 1 noted a concern about missing out on researchers who do not submit to the REF. In REF2021, all research active academics who were research independent were required to be submitted (in contrast to REF2014 where universities could choose not to submit some researchers). This ensured we had a comprehensive sample of researchers eligible for sabbaticals.

 Our selection approach focussed on researchers who were producing high quality research (measured both by publications, prizes and impact) stemmed from the wider project's desire to understand how discretionary (e.g., block grant) funding to universities supports high quality research. As Reviewer 2 notes, it is possible that we would have found different results had we selected our interviewees simply by looking for researchers who had recently had sabbaticals – we partially address this question in our quantitative analysis that looked at all researchers in the university who both published and took sabbaticals.

*Reviewer 1: On the sabbatical analysis - you do state that requests are largely automatically granted - is this really the case? did you speak to any unsuccessful applicants? (could be reflected on in limitations if data not available). The procedure at Cambridge seems quite different to other universities (where e.g. you are eligible to take them every 5 years, for up to 1/mo per year of service).*

*Cambridge is probably unusual in having departments and colleges. How does that complicate the picture?*

 We have reordered the paragraphs in the section on ‘Analysis of Sabbatical Uptake’ to make clearer that sabbatical practice differs between the departments of the university. There are departments where sabbaticals are generally not taken, however, in departments where they are taken, all of our interviewees suggested that they were approved as an administrative process.

 We are clear in the paper that our study focussed on Cambridge, and we have added an additional section on Limitations where we refer specifically to this and the implications. Making comparisons with other institutions was unfortunately not in the scope of our project. Cambridge certainly has more generous sabbatical arrangements than many other institutions, but we suspect that the college structure makes little difference to the practice of taking sabbaticals (although it may well have effects in other areas).

**Other demands on academics' time**

*Reviewer 1: It would also be good to reflect on the other demands on academics' time. They have a very different effect if you have a low teaching burden (which is often the case for more senior staff). I imagine this varies across the university?*

 In the study, academics generally highlighted the benefits of the uninterrupted time provided by sabbaticals, but did not, in general, distinguish between the types of activities that caused the interruptions or took up their time. We asked specifically about the teaching/research nexus which we discuss, but unfortunately don’t have a good source to allow us to reflect on the other demands on academics' time (except in the general sense in which we note the time provided by sabbaticals reduced these other burdens/interruptions).

**Wordsmithing suggestions/corrections**

*Reviewer 1: Slightly confusing language? " However – this effect appears to be largely due to the larger number of times researchers spend in their first (initial?) years after sabbatical, providing them more opportunities to publish at these times (as examined in the Methods section)." Would it be more accurate to say "at Cambridge, sabbaticals tend to be short and frequently taken, so the first year after sabbatical occurs frequently. Or something similar?*

*Reviewer 1: Typo? "When we normalised publication rate by dividing the publication rate by the number of times researchers spend in each period after sabbatical, the increases in publication post-sabbatical were no longer visible"*

 Reviewer 1 identified instances of confusing language and typos. These have been amended. We have taken the suggestion to use the word ‘initial’ and noted the implication of short sabbaticals, but not the comparative text about Cambridge sabbaticals being more frequent and shorter, as we don’t have the data to back up that assertion.

**Patterns across different demographics**

*Reviewer 1: I'd welcome more reflection on the points above, as well as on:What are the patterns across panels, age, career stage, and sex? What do you think might explain these patterns (given that you collated data on this, it would be good to know).*

 It would indeed be interesting to study whether there were differences in the ways sabbaticals were used across the different demographic characteristics we sampled. Our quantitative analysis did not discover any differences between disciplines and interviewees did not discuss this directly. We have added this to the new Limitations section of our paper, which we have added based on reviewers’ suggestions.

**Comparisons with other settings**

*Reviewer 1: I'd welcome more reflection on the points above, as well as on: As you say, comparison with other settings and policies.*

 Making comparisons with other settings and policies was unfortunately outside the scope of the project that was conducted. As mentioned in our new Limitations section, we think that a study that solely focussed on sabbaticals would be well placed to make such comparisons.

**Other measures of productivity**

*Reviewer 1:* *I'd welcome more reflection on the points above, as well as on: I like the idea of working papers as a measure of productivity. What about grant applications? Or research activities like conferences, roundtables, etc?*

 Reviewer 1 mentioned other interesting ways to capture research productivity during sabbaticals. We did attempt an analysis of grant application data – but were unable to clean the data to a sufficient extent. We have added these suggestions to the Conclusion section about future research directions.

**Wellbeing contributions of sabbaticals**

*Reviewer 1* *: I'd welcome more reflection on the points above, as well as on: What about the effect on wellbeing? (given that it came up in interviews).*

 We have made the explicit link between sabbaticals’ effect on wellbeing which was added following the second block quote in the sub-section ‘Summary of interviews on the teaching-research nexus’.

**Insight from interviews with ROO, HR, Finance**

*Reviewer 2: You mention you also consulted University administrators, including staff from the Research Office, HR personnel, and School Finance Managers. What did they say, and/or what contribution did they make to the project?*

 Reviewer 2 wanted to know what University administrators said in our interviews with them. These comments are woven into the Results section of the paper already and appear in the sub-section: ‘Analysis of sabbatical uptake’. Overall, these interviews told us more about the process of sabbatical taking – for example that it was largely administrative – but didn’t shed light on the ways in which sabbaticals were used.

**Structural points**

*Reviewer 2: Overall, the work is a little unclear in its presentation and would benefit from re-ordering and editing.*

*Introduction*

*While logic suggests there might well be a connection between sabbaticals and increased publication output, this is not mentioned in your introduction. This link should be explained and backed up with references, if they exist, or else it could be proposed as part of your hypothesis. This is particularly important as you go on to explain, in great detail, selection of interviewees on the basis of their publication outputs.*

*Somewhat related, I suggest this sentence, for example, belongs in the introduction: “Early in our project, our advisory group suggested the importance of investigating the interrelations between teaching, research and sabbaticals.”*

*Literature review*

*I suggest that this be a stand-alone section ahead of methods. The section on literature review currently in Results, which unnecessarily repeats information from the earlier section, could be brought into it.*

*Methods and Results*

*The Results sections, while very interesting, bears little relationship to the long explanations in Methods. I suggest the Methods should be amended to tie better to the reporting of the Results.*

 We appreciate Reviewer 1 and Reviewer 2’s suggestions about improving the links between the sections. We have added various changes which we hope make the links between sections clearer (many detailed in our earlier comments). The biggest change is to extract the Literature review into its own section preceding the Methods.

# Peer review: Reviewer response for version 1

**DOI:** 10.5256/f1000research.77940.r119613

**Published:** 2022-01-13

**Authors:** Kathryn Oliver

**Affiliations:** 1 Faculty of Public Health and Policy, London School of Hygiene and Tropical Medicine, London, UK

Thanks for the chance to review this interesting piece! Really worthwhile study. A few comments:

 You say the literature is very thin - can you provide some examples? It would also be useful in the initial paragraphs to clearly state what the questions are which you think these papers fail to address. e.g. (guessing) They fail to evaluate the cost of sabbaticals to universities, the impact on peers, careers, research productivity, teaching participation etc. 

 I see you excluded all the 1st person narratives about sabbaticals. Would these not help in contextualising your conclusions? (maybe not of course, but good to say clearly why not). 

 On the interviews: I don't fully understand why you took such a complicated sampling strategy for the 18 non-prize winners. Can you explain? I assume you wanted a process to enable you to generate a sample stratified along certain criteria? What might be the downside of your approach (I'm thinking that not everyone was selected for submission to the REF for instance). 

 On the sabbatical analysis - you do state that requests are largely automatically granted - is this really the case? did you speak to any unsuccessful applicants? (could be reflected on in limitations if data not available). The procedure at Cambridge seems quite different to other universities (where e.g. you are eligible to take them every 5 years, for up to 1/mo per year of service). 

 It would also be good to reflect on the other demands on academics' time. They have a very different effect if you have a low teaching burden (which is often the case for more senior staff). I imagine this varies across the university?

 Slightly confusing language? " However – this effect appears to be largely due to the larger number of times researchers spend in their first ( *initial*?) years after sabbatical, providing them more opportunities to publish at these times (as examined in the Methods section)." Would it be more accurate to say "at Cambridge, sabbaticals tend to be short and frequently taken, so the first year after sabbatical occurs frequently. Or something similar?

 Typo? "When we normalised publication rate by dividing the publication rate by the number of times researchers spend in each period after sabbatical, the increases in publication post-sabbatical *were *no longer visible"

 I'd welcome more reflection on the points above, as well as on:

- What are the patterns across panels, age, career stage, and sex? What do you think might explain these patterns (given that you collated data on this, it would be good to know).
- As you say, comparison with other settings and policies.
- Cambridge is probably unusual in having departments and colleges. How does that complicate the picture?
- I like the idea of working papers as a measure of productivity. What about grant applications? Or research activities like conferences, roundtables, etc?
- What about the effect on wellbeing? (given that it came up in interviews). 

Is the work clearly and accurately presented and does it cite the current literature?

Yes

If applicable, is the statistical analysis and its interpretation appropriate?

Yes

Are all the source data underlying the results available to ensure full reproducibility?

No source data required

Is the study design appropriate and is the work technically sound?

Yes

Are the conclusions drawn adequately supported by the results?

Yes

Are sufficient details of methods and analysis provided to allow replication by others?

Yes

Reviewer Expertise:

Evidence production and use

I confirm that I have read this submission and believe that I have an appropriate level of expertise to confirm that it is of an acceptable scientific standard, however I have significant reservations, as outlined above.
